# Distribution Change of Invasive American Bullfrogs (*Lithobates catesbeianus*) by Future Climate Threaten Endangered Suweon Treefrog (*Hyla suweonensis*) in South Korea

**DOI:** 10.3390/ani11102865

**Published:** 2021-09-30

**Authors:** Kyo Soung Koo, Minjee Choe

**Affiliations:** 1 Research Institute of EcoScience, Ewha Womans University, Seoul 03760, Korea; 2 EcoCreative Department, Ewha Womans University, Seoul 03760, Korea; minjeechoe@gmail.com

**Keywords:** amphibian, climate change, invasive species, Maxent, modeling

## Abstract

**Simple Summary:**

The American Bullfrog (*Lithobates catesbeianus*), known as one of the most problematic amphibians in the world, was introduced to South Korea in the 1970s. Although it has spread very rapidly over the past 50 years, there is no focused strategy to manage the frogs. With the introduction of American Bullfrogs into nature, numerous native species (mostly amphibians) are rapidly decreasing due to predation, a transmission of infectious diseases, and crossbreeding. In particular, the critically endangered Suwon treefrog (*Hyla suweonensis*) is the most affected by Bullfrogs. In this study, we modeled what environment the American Bullfrog settled in and how it could spread in the future. It was confirmed that the distribution of the two species overlaps in many areas at present, and the distribution of the American Bullfrog may spread to the distribution area of the Suwon treefrog according to future climate change. Through the results of this study, we intend to suggest a management direction for the spread of invasive species and the protection of endangered species. Our result could contribute to many countries that have problems with the American Bullfrogs at present and in the future.

**Abstract:**

The American Bullfrog (*Lithobates catesbeianus*) has been imported into South Korea in earnest for food since the 1970s and introduced into nature due to release and escape. Accordingly, the influx and spread of American Bullfrogs are expected to have a direct impact on native species, but few related studies have been conducted on this. We predicted changes in the potential distribution and future distribution based on climate change scenarios to analyze how those changes affect critically endangered Suwon treefrogs. Suwon treefrog sites (63.9%, 78/122) overlapped with the distribution of Bullfrogs. According to the prediction of the future distribution of Bullfrogs, the overlapping of American Bullfrogs and Suwon treefrog will remain similar to the current level in the Representative Concentration Pathway (RCP) 4.5 scenario. On the other hand, in the RCP 8.5 scenario, the number of overlapping sites will increase to 72.1% (88/122) due to the spreading of the American Bullfrogs. The results show that climate change directly affects the distribution expansion of the American Bullfrogs but also indirectly can lead to an increased threat to Suwon treefrogs. In conclusion, our results strongly suggest why climate change should be actively addressed in terms of the spread of invasive species and the protection of endangered species.

## 1. Introduction

The decline in biodiversity is caused by many reasons such as habitat destruction, pollution, climate change, and disease. Recently, invasive non-native species have become a threat to ecosystem biodiversity as globalization facilitates their spread. The invasion of the non-native species is caused by natural reasons such as habitat changes due to climate change, accidental draft, or typhoons. On the other hand, the artificial introduction is also one of main causes of invasion, such as with pest controls [1], food [2], pets [3,4], and religious release [5]. In many cases, the introduced species die out naturally, but once they settle successfully, it is difficult to remove them by artificial methods. The non-native species that are introduced to the wild not only cause competition [6], predation [7], and hybrid formations [8] with the endemic species but also problems over the wider area such as parasites or the inter-mediation of diseases [9,10]. Moreover, the socio-economic damages required to control the invasion of non-native species are damages that humans directly suffer from [11,12].

The American Bullfrog (*Lithobates catesbeianus*) is native to the United States and Canada and is one of the most widespread amphibians in the world [13,14]. At a size of over 150 mm, the American Bullfrog is advantageous for the predation of small endemic species, and the characteristic of laying more than 20,000 eggs facilitates the rapid formation and spread of the population when settled in a new environment [15,16]. In South Korea, the American Bullfrog was first imported in late 1950 for the purpose of edible usage, but their attempted cultivation failed [5]. Later, they were re-imported from Japan in 1973, and after successful farming, spread to farms across the country. However, in most cases, low profits and decreases in consumption due to dietary changes have increased the number of farms that have given up farming, and most of the imported species were released into the wild [17]. Over the past 50 years, American Bullfrogs have been found in very large regions, including many islands [17]. The American Bullfrog in South Korea has food sources such as endemic species, birds, frogs, insects, and small mammals including endemic and endangered turtles such as *Mauremys reevesii* [18,19,20]. In addition, previous studies have shown that the decrease in the seriously endangered Suwon treefrogs (*H. suweonensis*) may be linked to diseases mediated by *L. catesbeianus* [10]. Therefore, the spread of *L. catesbeianus* has resulted in an additional problem of ecological disturbances in South Korea, and it has the possibility to continue in the future.

The study on species distributions through modeling is instrumental in predicting settlement and spread after species introduction [21]. The invasion of the *L. catesbeianus*, a representative non-native species, has been the subject of several regional distribution predictions [22,23,24] and distribution studies conducted around the world [21]. A prior study predicted that *L. catesbeianus* could be distributed with high probability throughout the country, and the actual distribution accorded with that prediction except in some inland areas [17]. In addition, the diseases spread by *L. catesbeianus* have been estimated by modeling methods [25]. Modeling also makes available the ability to predict the effects among interspecies or intergroup through changes in the distribution of organisms. The distribution modeling is crucial to predicting the distribution of species that are currently coming into the country, as well as those with the potential of entering into the country, and to the prediction for the distributions and the possible spread of mediated diseases [25,26]. Using modeling to determine the distribution characteristics of an organism is a fundamental criterion for setting the range for control of the target species.

*Hyla suweonensis* is an endemic species that was first discovered in 1980, which is very similar to the Japanese treefrog (*Hyla japonica*) but has distinctly characterized calling and genetic traits [27,28]. This endemic species is usually found only in rice paddies and has the characteristic of hibernating around rice paddies [29,30]. However, the number of species is decreasing rapidly as the area of rice paddies diminishes [31]. Currently, they are only found in limited areas in the west part of Korea [32]. In addition, it is believed that the propagation of chytrid fungus (*Batrachochytrium dendrobatidis*) by *L. catesbeianus* will be a serious threat to *H. suweonensis* [10,32]. As a result, the Korean government has designated *H. suweonensis* as endangered species (Class 1) and has included them in the red list to protect them. So far, many research results have been announced to protect them, but it is difficult to find specific management methods and scope related to *L. catesbeianus*, a direct threat factor.

In this study, we predicted the potential distribution of *L. catesbeianus* in South Korea according to climate change scenarios. Additionally, we assessed how the change in the distribution of *L. catesbeianus* would affect endangered *H. suweonensis*. It will also be the basis for effective control of *L. catesbeianus* that have been introduced to South Korea and are rapidly spreading, and the basis for establishing strategies for the protection of seriously endangered *H. suweonensis*.

## 2. Materials and Methods

### 2.1. Collecting Location Data

The location data used for the prediction of distribution and spreading model is based on the database from the previous study [17]. The location data used in prior research are from the four-time repeated state-led surveys for introduced species covering the entire region of South Korea from 2006 to 2017 [17]. In order to reduce the differences in results according to the investigation method, experts in the field of amphibians conducted it based on the same survey guidelines [33]. To minimize the redundancy for the site survey, we divided the entire country’s land (824,155.7 km^2^) into the grid units (1 cell: 11.2 km × 13.9 km) [17]. To reduce errors or redundancy in distribution data, we checked each point through Google Maps and QGIS (2020) and retained or maintained a total of 2416 spots [17,34]. The distribution of *H. suweonensis* was based on a survey of potential habitats from 2014 to 2016, and distribution data of the 122 locations that directly identified the objects used in this study [35].

The climate variable made use of the 19 bioclimatic variables from 1960 to 1990 with 30 arc-second grids (~1km) provided by Worldclim (ver 1.4. http://www.worldclim.org; accessed on 9 September 2020) [36]. Spearman’s correlations analysis was used to reduce the collinearity that causes similarities among the variables [25]. Similar variables were removed on the basis of the correlation r > 0.7 between the two variables. The results showed that a total of five variables were suitable for predicting the distribution of *L. catesbeianus* in Korea: Isothermality (Bio03), Maximum Temperature of the Warmest Month (Bio05), Minimum Temperature of the Coldest Month (Bio06), Annual Precipitation (Bio12), and Precipitation of the Wettest Month (Bio13) (Table 1). The variables, Bio01 (Annual Mean Temperature), Bio02 (Mean Diurnal Range), Bio04 (Temperature Seasonality), Bio07 (Temperature Annual Range), Bio08 (Mean Temperature of Wettest Quarter), Bio09 (Mean Temperature of Driest Quarter), Bio10 (Mean Temperature of Warmest Quarter), Bio11 (Mean Temperature of Coldest Quarter), Bio14 (Precipitation of Driest Month), Bio15 (Precipitation Seasonality), Bio16 (Precipitation of Wettest Quarter), Bio17 (Precipitation of Driest Quarter), Bio18 (Precipitation of Warmest Quarter), and Bio19 (Precipitation of Coldest Quarter), were removed due to collinearity (see Hijmans et al. 2005 for the details of variables) [37].

For the climate change scenario, HadGEM2-ES (HE), the scenario that was used in the field of animals including the field of amphibians among the global climate models (GCMs), was used [36,38,39]. The GCMs provide four Representative Concentration Pathways (RCPs) according to changes in greenhouse gases; RCP 2.6: 2.6 W/m^2^, RCP 4.5: 4.5 W/m^2^, RCP 6.0: 6.0 W/m^2^ and RCP 8.5: 8.5 W/m^2^ [40,41]. The RCP 4.5 and RCP 8.5 scenarios for the modeling were used in this report, the scenarios where the greenhouse gas emissions are maintained as of now, and the case that they are in the most critical condition [41]. For the prediction of future potential distribution, the data indicating the near future and far future were used [37].

Unlike the data of *L. catesbeianus* obtained by repeatedly surveying the country’s entire land, the case of *H. suweonensis* is based on an optional survey of the expected areas [35]. Therefore, it was determined that the data was not enough to predict potential and future changes in distributions of *H. suweonensis.* The impact of the change in the distribution of *L. catesbeianus* was determined by utilizing only the spot where *H. suweonensis* was found.

### 2.2. Predicting Present and Future Distribution

In the case of the data we have used, it is the result of repeated surveys of a wide range of regions, but data on absence are not included. Therefore, we used the Maxent (ver 3.4.1, American Museum of Natural History, New York, NY, USA), a species distribution model, to predict species’ distribution based on presence data [42]. To predict the future distribution of *L. catesbeianus*, we used a time period (2070, average 2061−2080) where 75% of the distribution data were randomly selected and used as training data for modeling. The remaining 25% of them were used to test the generated model. The deviation between models occurs because we randomly selected the distribution data and derived the results of the model. To reduce such deviation, each model was run 10 times and the average value of the models was used. The accuracy of the model was indicated as the AUC (area under the ROC (receiver operating characteristic) curve) value [43]. Usually, the AUC value is expressed as 0.5–1, and the model is more reliable as the value is closer to 1 [40].

## 3. Results

### 3.1. Potential Distribution of Lithobates catesbeianus

**Figure 1:**
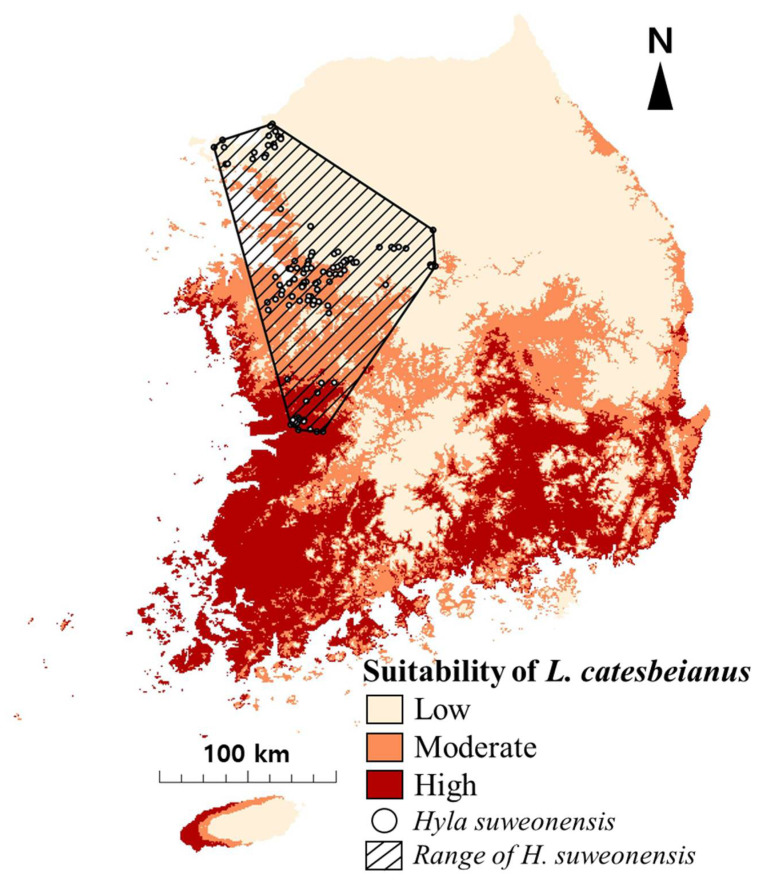
The potential distribution of *Lithobates catesbeianus* and the actual distribution points of *Hyla Suweonensis* in South Korea.

The AUC for the potential distribution model of *L. catesbeianus* was 0.808. The variables that mainly affect the distribution were minimum temperature of the coldest month and the maximum temperature of the warmest month, and the contributions to the creation of models were 57.4% and 33.4%, respectively (Table 2). It turned out that the distribution of *L. catesbeianus* should be concentrated in the southern part of the country (Figure 1).

The distribution of *H. suweonensis* was mainly concentrated in the western part of the country. A total of 78 spots (63.9%) were included within the potential distribution range of *L. catesbeianus*, excluding some parts of the northwest and inland (36.1%, 44 spots) (Figure 1, Table 3).

### 3.2. Future Distribution of Lithobates catesbeianus

**Figure 2:**
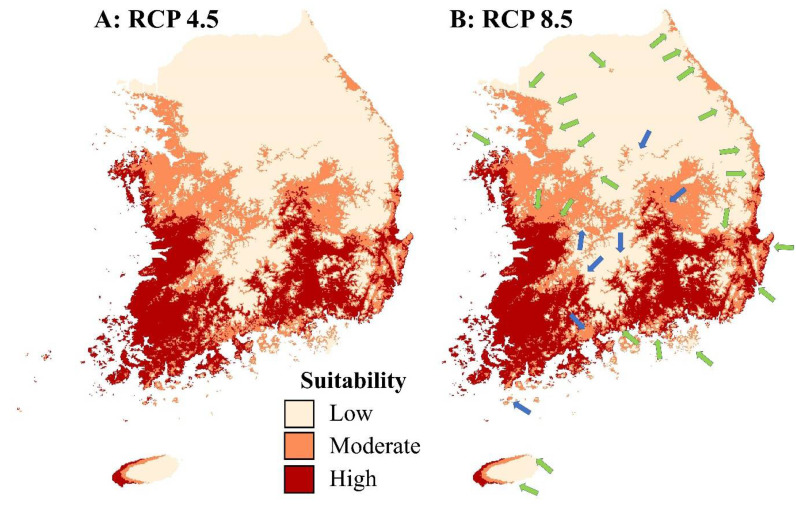
Changes in the distribution of *Lithobates catesbeianus* in the climate change scenarios. (**A**) RCP 4.5, (**B**) RCP 8.5. The arrows indicate the increase (green) and decrease (blue) of predicted distribution in the future.

The reliability of the model predicting the future distribution shows that AUC is 0.800–0.802. The most significant influential factor in the future distribution of *L. catesbeianus* was Bio 06 (Min Temperature of Coldest Month), which was the same in all scenarios (Table 2). The distribution of *L. catesbeianus* was expected to increase mainly in the western, eastern, and southern coastal areas, and decrease inland and some islands (Figure 2A,B).

**Figure 3:**
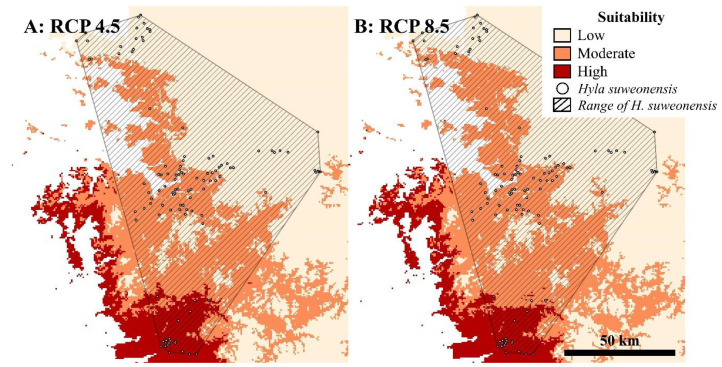
Overlapping with the distribution of *H. suweonensis* with the distribution of *Lithobates catesbeianus* in each scenario. (**A**) RCP 4.5, (**B**) RCP 8.5.

The distribution spots of *H. suweonensis*, which overlaps with future *L. catesbeianus*, decreased from 81 spots to 79 spots in the RCP 4.5 scenario (Table 3). However, the number of overlapped spots between the distributions of the two species is expected to increase under the RCP 8.5 scenario (Figure 3A,B).

## 4. Discussion

The potential distribution of *L. catesbeianus* is wider than the actual area where it is found and is expected to spread further in the future [22,24,44]. The reason is that future climate changes, especially temperature changes, will expand the range of survivable habitats and be a decisive factor in the spread of *L. catesbeianus*. Even in South Korea, the most significant climatic factor affecting the distribution of the species was predicted as winter temperatures. South Korea has four distinct seasons, and the temperature in winter drops below zero. Thus, the distribution of *L. catesbeianus* was concentrated on relatively warm coasts (west, south, and east). However, according to the future climate change scenarios, the range of areas where they can survive will increase as the winter temperature increases. Eventually, considering the current climate change, it is difficult to expect a natural decline in *L. catesbeianus* populations. Thus, we need more active and artificial ways to control the spread.

*H. suweonensis* has a small scope of behavior and does not move much between breeding and habitat (brumation) places [30]. This trait may cause a lower chance of avoidance when *L. catesbeianus* is introduced. The case of a direct preying on *H. suweonensis* has not been reported so far, but there has been a reported case that *H. japonica*, a very closely related species of *H. suweonensis*, is a prey [7]. In addition, infectious diseases mediated by *L. catesbeianus* can also be a potential problem for *H. suweonensis* [10]. Consequently, considering our results that the distribution of *L. catesbeianus* can be expanded, the damage on *H. suweonensis* is likely to become more severe. Based on the current model results, we suggest that areas where the two species are overlapped or are possible to be overlapped should be set as protected areas in advance. The policy approach, which applies the results of these studies, will be an essential task that should be preceded to protect endangered species that are rapidly decreasing.

The species distribution modeling is critical in predicting the distribution of introduced species. However, the result can be distinguished according to the local environment, the adaptability of a species, and the data used [21]. For example, research by [21,22], which predicted the potential distribution of *L. catesbeianus* in the United States, used the same species distribution modeling program, Maxent, but there were differences in the habitat suitability. These differences can be seen as the results of limited monitoring, the scope of the study, the number of distribution spots used, variables, and settings used for modeling [21,36]. As another example shows, significant differences in the spot number and the distribution number of *L. catesbeianus* were recorded in two papers [7,17] published in the same year in South Korea. If distribution spots and regions are not enough to be used in the modeling for representing the area, the estimates are likely to be underestimated or overestimated [7]. Moreover, the environmental variables that contributed to predicting the potential distribution of *L. catesbeianus* were also different in each study because the differences in the variables according to the study were the result of considering multicollinearity and so forth [21,22,44]. This difference can be seen as the result that the distribution characteristics of *L. catesbeianus* in each region are reflected well. In summary, it means that a sufficient number of distribution data obtained within the scope, in which the characteristics of the distribution of lives can be enough to be reflected in the target area, should be requested.

## 5. Conclusions

Our results indicate that *L. catesbeianus*, which about 50 years ago was introduced to South Korea, has spread widely and will continue to spread further in the future. This expansion will not only disturb the ecosystem but also affect the native species including *H. suweonensis*. It is a big challenge internationally and regionally to control *L. catesbeianus* that has completely adapted to new environments. However, we need to closely monitor and study adaptive aspects continuously for the full control of *L. catesbeianus*. It is also necessary to establish specific management methods and scopes, considering the relationships with endemic species, including endangered species.

## Figures and Tables

**Table 1:** Bioclimatic variables on the distribution of *Lithobates catesbeianus* in South Korea.

Bioclimate Variables		Mean	Min	Max
Bio03	Isothermality (BIO2/BIO7) (×100)	27.8	22.0	33.0
Bio05	Max Temperature of Warmest Month (°C)	30.0	27.0	31.0
Bio06	Min Temperature of Coldest Month (°C)	−4.8	−11.1	2.5
Bio12	Annual Precipitation (mm)	1263.6	1003.0	1637.0
Bio13	Precipitation of Wettest Month (mm)	256.5	172.0	416.0

**Table 2:** The percentage of contribution for species distribution modeling on *Lithobates catesbeianus* introduced in South Korea. Present: 1960–1990, Future: 2070 (2061–2080) (Hijmans et al. 2005) [37].

Bioclimate Variables	Contribution for Modeling (%)		
	Present	Future	
		RCP 4.5	RCP 8.5
Bio03	1.5	2.7	1.3
Bio05	33.4	31.1	27.6
Bio06	57.4	56.0	63.3
Bio12	3.4	3.2	2.2
Bio13	4.3	7.0	5.7

**Table 3:** The number of overlapping points with the distribution of the *Hyla Suweonensis* according to the change in the distribution of *Lithobates catesbeianus*.

Potential Distribution	Number of Overlap Points	
	RCP 4.5	RCP 8.5
Present	78/122 (63.9%)	
Future	79/122 (64.8%)	88/122 (72.1%)

## Data Availability

Not applicable.
